# Novel regulation on the mitotic checkpoint revealed by knocking out *CDC20*

**DOI:** 10.3389/fcell.2023.1276532

**Published:** 2023-10-02

**Authors:** Ying Wang, Yuqing Zhang, Gang Zhang

**Affiliations:** ^1^ School of Public Health, Qingdao University, Qingdao, China; ^2^ The Cancer Institute, The Affiliated Hospital of Qingdao University, Qingdao University, Qingdao, China

**Keywords:** CRISPR, Cdc20, SAC, CRY box, translation reinitiation

## Introduction

To ensure accurate segregation of genetic materials during cell division, eukaryotic cells employ a highly conserved signaling pathway called the spindle assembly checkpoint (SAC) to monitor the generation of a stable attachment between microtubules and kinetochores. Unattached kinetochores activate the checkpoint by recruiting multiple checkpoint proteins, which further catalyze the formation of the mitotic checkpoint complex (MCC). Composed of BubR1, Bub3, Mad2, and Cdc20, MCC directly binds to and inhibits the anaphase-promoting complex/cyclosome (APC/C), the E3 ligase responsible for the ubiquitination of key mitotic regulators like cyclin B1 and securin. High Cdk1/cyclin B1 kinase activity retains the cells in mitosis; thus, the cells are given more time to generate a stable kinetochore–microtubule attachment. Once the stable attachment is generated, the checkpoint is silenced from the kinetochores and the produced MCC is disassembled via multiple mechanisms. Cyclin B1 and securin gets degraded, and cells enter anaphase with coordinated chromatid segregation (Musacchio, 2015; Lara-Gonzalez et al., 2021; McAinsh and Kops, 2023). However, even in the persistence of an active checkpoint, cells could still exit mitosis and enter interphase without proper chromosome segregation and cytokinesis. This unusual mitotic exit is called mitotic slippage and yields tetraploid multinucleated cells which could either die after the slippage or arrest in the G1 phase, or continue the cell cycle. Mitotic slippage could be caused by the gradual degradation of cyclin B1 by the leakage activity of APC/C or by the weakening of SAC (Topham and Taylor, 2013). As a key mitotic regulator, Cdc20 plays opposite roles in mitosis as one molecule binds and activates APC/C to promote anaphase initiation and another molecule forms MCC to inhibit APC/C^Cdc20^ and delay anaphase initiation (Izawa and Pines, 2015; Alfieri et al., 2016). Although Cdc20 has been extensively studied, its functional investigation within cells is limited by the fact that mammalian cells only need a minimal amount of Cdc20 for cell division and that RNAi against Cdc20 could not efficiently deplete the protein to induce a penetrant phenotype (Wolthuis et al., 2008; Baumgarten et al., 2009). Meanwhile, complete inactivation of *CDC20* causes metaphase arrest, followed by apoptosis; thus, a genuine knockout cell line could not be generated (Li et al., 2007).

CRISPR/Cas9-mediated gene knockout has been widely applied in gene function analysis. By introducing DNA fragment insertion or deletion (indels), the targeted gene is disrupted as the reading frame is shifted and a premature termination codon (PTC) occurs (Doudna and Charpentier, 2014). Until now, numerous knockout cell lines or organisms have been generated, which significantly promoted the gene function investigation. However, mounting pieces of evidence show that disrupting the reading frame may not fully silence the targeted genes. Truncated proteins could be produced via two distinct mechanisms to partially or fully compensate the putative loss of function. Translation reinitiation utilizes the inframe AUG downstream of the original start codon to produce N-terminal truncated proteins, while alternative splicing generates internally altered proteins (Smits et al., 2019; Tuladhar et al., 2019; Zhang et al., 2019). These proteins may still maintain some activity of the wild-type protein as far as the essential domains remain intact. Since the amount of these truncated proteins is generally lower than the wild-type protein and the epitopes recognized by the antibodies might be disrupted, their presence is often neglected, resulting in an imprecise phenotype interpretation. On the other hand, it also provides convenient methods to study the essential genes which are difficult to be silenced by RNAi alone, as demonstrated previously (Zhang et al., 2019; Wang et al., 2023). Here, we comment on the two new studies revealing novel SAC regulatory mechanisms by knocking out the essential *CDC20* (Tsang and Cheeseman, 2023; Zhang et al., 2023).

## Alternative translation of *CDC20* promotes mitotic slippage

*CDC20* has been assumed to express a single protein of approximately 55 kDa. However, this was challenged by the discovery of two smaller Cdc20 protein variants from a panel of cell lines using an antibody against the C-terminus of Cdc20 (Tsang and Cheeseman, 2023). Eliminating the start codon or introducing a frame shift within exon 1 by CRISPR/Cas9 fully abrogated the full length protein but not the smaller variants, indicating translation reinitiation occurred. Indeed, further analysis showed the two Cdc20 variants were generated from downstream translation start sites at positions 43 and 88 (referred to as M43 and M88 hereafter). Probably due to the lack of the Mad1-binding motif (BM1, 27–34aa), the two variants cannot support SAC further. Cdc20 M88, but not M43, loses the APC/C-binding motif (C box, 77–83aa) and cannot activate APC/C. Interestingly, the lack of a functional mitotic checkpoint does not affect the cell viability. Since the mitotic checkpoint only becomes dispensible when the APC/C activity is weakened (Wild et al., 2016), we speculate that the APC/C activity is also attenuated and a new balance is achieved in these cells.

**FIGURE 1:**
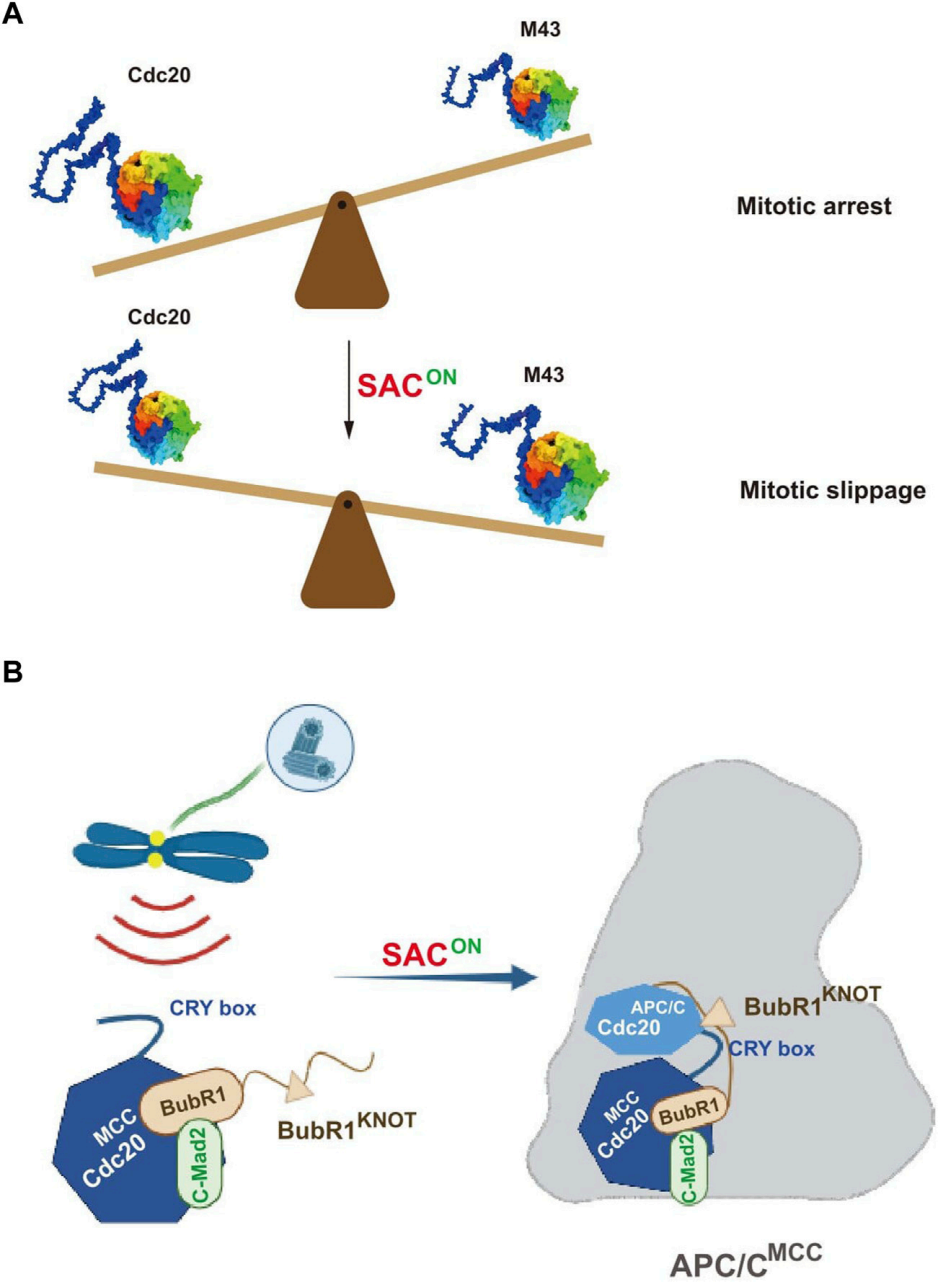
Pictorial representations showing the novel regulation on the mitotic checkpoint by Cdc20. **(A)** Fine tuning of the mitotic arrest duration by an alternatively translated Cdc20 isoform, M43. During the mitotic arrest by the activation of SAC, the ratio between wild-type Cdc20 and the SAC-resistant isoform M43 decreases in favoring the mitotic slippage. **(B)** Cdc20 CRY box is critical for SAC-mediated APC/C inhibition. The CRY box is required for both MCC formation and the interaction between MCC and APC/C. Disruption of the CRY box causes mitotic slippage in the presence of microtubule poisons (adapted from Zhang et al., 2023).

The authors further examined the effect on SAC by altering the expression levels of the Cdc20 variants, especially Cdc20 M43. Overexpression of Cdc20 M43, but not the full-length protein, significantly promoted mitotic slippage even in the presence of the checkpoint activator. More importantly, the ratio between the full-length Cdc20 and M43 decreased gradually during the mitotic arrest when SAC was on. It is thus assumed that once the relative ratio of Cdc20 M43 increased to a certain level, it could efficiently compete with the full-length Cdc20 to activate APC/C, which further induces mitotic slippage. Thus, an appropriate mitotic timer is proposed to tune the duration of mitotic arrest in favor of cell survival (Figure 1A). Whether this mechanism also plays a role for unperturbed mitosis awaits further investigation.

## The Cdc20 CRY box is critical for SAC activation

It is estimated that the endogenous Cdc20 protein level has to be reduced to less than 5% for the cells to show mitotic arrest (Wolthuis et al., 2008; Baumgarten et al., 2009). To precisely characterize the function of each motif on Cdc20 in a background without the interference by a residual endogenous protein, we also generated *CDC20*-knockout HeLa cells with the help of CRISPR/Cas9 (Zhang et al., 2023). In these cells, no Cdc20 was detected by the antibody against the C-terminus. The viable knockout cells also could not activate SAC and spend longer time in unperturbed mitosis, indicating a weakened APC/C activity, likely due to the very low level of the residual Cdc20 protein. Indeed, the residual Cdc20 protein was detected by mass spectrometry. More importantly, a penetrant metaphase arrest could easily be achieved by RNAi with the knockout cells but not with the parental cells.

Based on this full knockdown background, we systematically measured the activity of APC/C and SAC with a series of Cdc20 mutants lacking individual motifs. We found the mutants with disrupted APC/C-binding motifs cannot activate APC/C further. Without treatment of microtubule poisons, cells expressing Cdc20△C box, Cdc20△IR, or Cdc20△KILR were all arrested at metaphase that lasted for more than 500 min and died afterward, demonstrating that all these motifs are required for activating APC/C. Unexpectedly, the CRY box, an unconventional degron of Cdc20 (Reis et al., 2006), was found to be critical for activating SAC. To understand the mechanism, we carefully analyzed the dissolved structure of MCC-APC/C (Alfieri et al., 2016) and found multiple potential interactions between Cdc20^MCC^, Cdc20^APC/C^, and BubR1^KNOT^ mediated by the CRY box of Cdc20^MCC^. Functional analysis showed that disrupting these interactions individually, indeed, impaired the checkpoint in cells, which corroborated the structural analysis (Figure 1B). Since the core sequence of the CRY box is highly similar to the C box, we also found the two motifs are functionally exchangeable. Future study is needed to understand the cooperation between these newly discovered interactions and previously identified interactions for the stable binding of MCC with APC/C.

## Discussion

By targeting *CDC20* via CRISPR/Cas9, two recent studies revealed novel regulation on SAC. In both studies, the *Cdc20*-knockout cell lines are generated. If only judged by the disappearance of the 55-kD band of Cdc20, researchers could easily make an incorrect conclusion that *CDC20* is not essential for cell viability. The viability of the knockout cells is supported by partially active Cdc20 truncates produced via translation reinitiation or alternative splicing. Based on these cells, Tsang and Cheeseman discovered a fine tuning mechanism of the SAC-induced mitotic arrest. We found a cryptic degron of Cdc20 is actually critical for SAC activation. Although the two studies focused on the mitotic checkpoint, we believe the strategy could be useful in the gene function investigation, especially the essential ones which are difficult to be silenced by RNA interference alone.
